# Circulating Irisin Levels in Patients with Nonalcoholic Fatty Liver Disease: A Systematic Review and Meta-Analysis

**DOI:** 10.1155/2020/8818191

**Published:** 2020-11-07

**Authors:** Jie Hu, Yani Ke, Fangping Wu, Shan Liu, Conghua Ji, Xiaohong Zhu, Ying Zhang

**Affiliations:** ^1^Department of Infectious Disease, The First Affiliated Hospital of Zhejiang Chinese Medical University, Hangzhou, 310006 Zhejiang Province, China; ^2^The Second Clinical Medical College of Zhejiang Chinese Medical University, Hangzhou 310053, Zhejiang Province, China; ^3^Department of Clinical Evaluation Center, The First Affiliated Hospital of Zhejiang Chinese Medical University, Hangzhou, 310006 Zhejiang Province, China; ^4^Department of Medical Record, Guangdong Women and Children Hospital, Guangzhou, 511400 Guangdong Province, China

## Abstract

**Background and Aims:**

Previous studies have revealed the close relation of irisin with the occurrence and development of nonalcoholic fatty liver disease (NAFLD). A systematic review and meta-analysis were conducted to evaluate the association of circulating irisin levels and NAFLD.

**Methods:**

A systematic literature search of PubMed, Embase, Cochrane Library, Clinicaltrials.gov, WANFANG, CNKI, and CBM databases was performed for relevant articles till August 2020. The weighted mean difference (WMD) values and 95% confidence intervals (CIs) were estimated to compare the case-control studies and pooled results using meta-analysis.

**Results:**

The meta-analysis included 5 case-control studies with a total of 1087 people. The results revealed that the circulating irisin levels showed no significant difference between NAFLD and healthy groups (WMD = 7.51 (-12.53, 27.56) ng/ml, *P* > 0.05). Subgroup analysis based on races showed that the average irisin levels were higher in the NAFLD group than in the healthy group (WMD = 13.53 (0.71, 26.34) ng/ml, *P* < 0.05) in 4 Asian studies. Subgroup analysis based on disease severity from 3 Asian studies revealed that the average irisin levels were higher in the NAFLD group than in the healthy group (WMD = 25.1 (22.85, 27.51) ng/ml, *P* < 0.05 and WMD = 13.52 (22.85, 27.51) ng/ml, *P* < 0.05, respectively). Subgroup analysis including 3 studies from Asia suggested that the irisin levels were higher in mild NAFLD than in moderate-severe NAFLD (WMD = 11.68 (9.03, 14.32) ng/ml, *P* < 0.05).

**Conclusion:**

The average irisin levels might be higher in the NAFLD group than in the healthy group in Asians. The irisin levels in the mild NAFLD group might be higher than those in the moderate-severe group in Asians. It is important to monitor the changing trend of irisin levels in predicting the course of NAFLD disease and its changes.

## 1. Introduction

Nonalcoholic fatty liver disease (NAFLD) is a metabolic liver injury that is characterized by insulin resistance and genetic susceptibility. The disease includes nonalcoholic fatty liver (NAFL), steatohepatitis (NASH), hepatic fibrosis, and cirrhosis [1]. The main pathological changes of this disease were hepatocyte steatosis and abnormal accumulation of fat in the hepatocytes. Currently, NAFLD has become a common liver disease throughout the world [2, 3] and is closely related to high incidence of metabolic syndrome, type 2 diabetes mellitus, and other diseases [4]. However, the specific pathogenesis of NAFLD is not completely clear. The prevalence of NAFLD is increasing year by year and presented low tendency with age, seriously endangering human health and causing huge social as well as economic burden.

Recently, Bostrom et al. [5] have found a membrane protein that is encoded by the type III fibronectin domain 5 (FNDC5) gene in the skeletal muscle, and this belonged to N-glycosylated protein hormone. They were the first to find that irisin was mainly produced by exercise in both mice and humans, and a small amount of irisin could be synthesized from the liver or secreted from the adipose tissue [5, 6]. Irisin improved the metabolism of sugars, fats, and amino acids by increasing energy consumption in the body, reversed liver steatosis, and improved insulin sensitivity. Irisin played an important role in the occurrence and development of NAFLD, but its mechanism is still not fully understood. The correlation between irisin and NAFLD has become a new research hotspot in the recent years, but the data of serum irisin in human NAFLD still remains to be controversial.

At present, many scholars have studied the levels of irisin in NAFLD patients all over the world, but the sample size of many studies is small, leading to inconsistent results. To our knowledge, there is no meta-analysis on the relationship between NAFLD and circulating irisin levels. Therefore, a systematic evaluation of the relationship between NAFLD and circulating irisin levels was conducted in this study to evaluate the association between the severity of the disease and circulating irisin levels in NAFLD comprehensively and to obtain potential therapeutic targets for NAFLD.

## 2. Materials and Methods

### 2.1. Literature Search

This systematic review was performed according to the standards of the Preferred Reporting Items for Systematic Reviews and Meta-Analyses (PRISMA) criteria [7] and the recommendations of the Cochrane Collaboration [8]. A protocol for this systematic review has been published in PROSPERO with the registration number CRD 42019130962. The published protocol is in Supplementary Materials (Supplemental File 1).

A systematic search of 7 databases (PubMed, Embase, the Cochrane Library, ClinicalTrials.gov, CNKI, WANFANG, and CBM) was performed by two authors for relative studies published till August 3, 2020. The search terms/MeSH terms used were “NAFLD”, “Nonalcoholic fatty liver disease”, “Non-alcoholic fatty liver disease”, “Nonalcoholic fatty liver disease”, “NASH”, “Non-alcoholic steatohepatitis”, “Nonalcoholic steatohepatitis”, “Nonalcoholic steatohepatitis”, “Fatty liver”, “irisin”, “FNDC5”, “fibronectin type III domain containing protein 5”, “Fndc5 protein”, and “FRCP2 protein”, and the full search strategy is detailed in Supplementary Materials (Supplemental File 2). The references of the included articles were searched for any other potential studies of the topic. No language restrictions were imposed. The authors were informed to collect the missing data.

### 2.2. Study Selection

The titles and/or abstracts of the studies were checked independently by two team members to identify relevant studies that met the inclusion criteria. The full texts of these potentially eligible studies were retrieved and independently assessed for eligibility. Any disagreements between the reviewers with regard to the eligibility of studies were resolved by discussing with a senior reviewer. A standardized, preformatted Excel form was used to extract the data from the included studies for assessing the study quality.

The following were the study selection criteria: (1) studies that included adults (aged 18 years or older) with NAFLD (NAFL or NASH) diagnosed either by imaging or by histology—the mild hepatic fatty degeneration and moderate-severe hepatic fatty degeneration were distinguished based on B-ultrasound results [9]; (2) case-control studies that included healthy or lean individuals as controls; (3) studies with the outcome of measurement of circulating irisin levels by the enzyme-linked immunosorbent assay (ELISA) in either plasma or serum; and (4) case-control studies or cohort studies that reported data on circulating irisin levels in individuals with NAFLD.

Exclusion criteria were as follows: (1) studies that included secondary hepatic fat accumulation, such as significant alcohol consumption, use of steatogenic medication, or hereditary disorders, and other known causes of liver diseases, e.g., virus and drugs; (2) studies published as reviews, meta-analysis, protocols, comments, editorials, basic science, or animal studies; (3) duplicate studies or studies with overlapping data sets or with the same study populations; and (4) studies with no relevant data obtained even by contacting the authors.

### 2.3. Data Extraction

Two researchers have independently extracted the following data (Table 1) including the first author's last name, publication date, country of origin, the Newcastle-Ottawa Scale (NOS), numbers of cases and controls, irisin level-measuring method, and levels of irisin. Any discrepancies between the two reviewers were resolved by reaching a consensus.

### 2.4. Risk of Bias

Publication bias was conducted using Egger's test [10]. A sensitivity analysis was conducted to evaluate the stability of outcomes by sequential omission of individual studies [11].

### 2.5. Statistical Analysis

All statistical analyses were performed using Stata 15 (Stata Corporation, TX). Continuous data were reported as weighted mean difference (WMD). The pooled WMD and the 95% confidence intervals (CIs) were determined by the *Z* test for assessing the difference between the groups. A *P* value and *I*^2^ test were used to identify heterogeneity. *P* values of more than 0.10 reveal no statistically significant heterogeneity, and so a fixed effects model (shown as “I-V”) [12] was used. *P* values of less than 0.10 means that significant heterogeneity was observed in the pooled results, and so a random effects model (shown as “D+L”) was used [13]. When the *I*^2^ value was greater than 50%, then subgroup analysis and sensitivity analysis were performed to explore the sources of heterogeneity. Subgroup analyses were performed by ethnicity and severity of NAFLD. The quality of included articles was evaluated using the NOS scale [14].

## 3. Results

### 3.1. Study Selection

**Figure 1:**
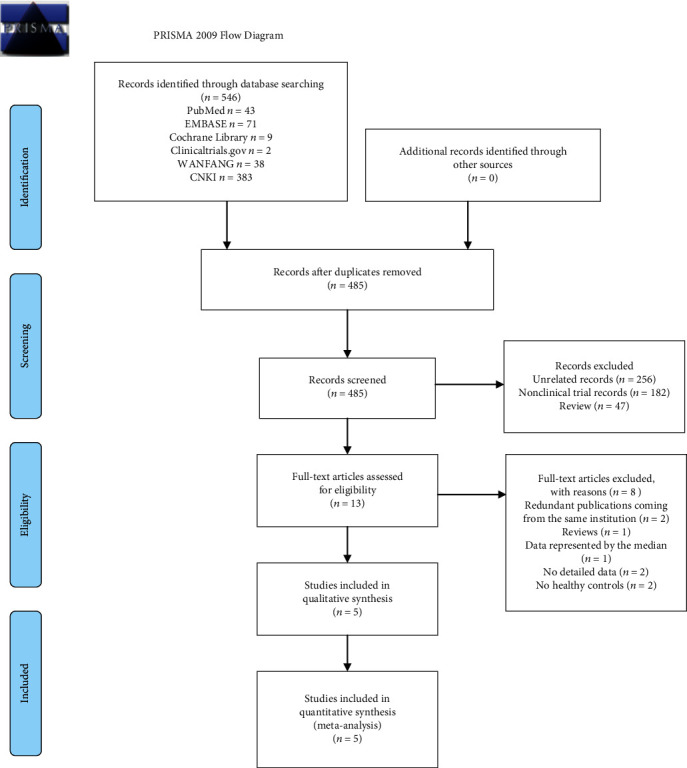
Flowchart of study inclusions and exclusions.

This study was conducted based on the PRISMA guidelines. A total of 421 articles in Chinese and 125 in English were retrieved (Figure 1). After removing the duplications, 398 Chinese articles and 87 English articles were screened. Thirteen studies were identified after reading the titles and abstracts. Finally, 5 studies including 434 cases and 653 controls were analyzed. All the 5 included studies and their main characteristics are presented in Table 1.

### 3.2. Quality Assessment

The average NOS score was 5.4, which meant that the quality of methodology was average. The score of 3 studies [15–17] was 5, and the remaining studies [18, 19] had a score of 6 (Table 2).

### 3.3. Irisin Levels

**Figure 2:**
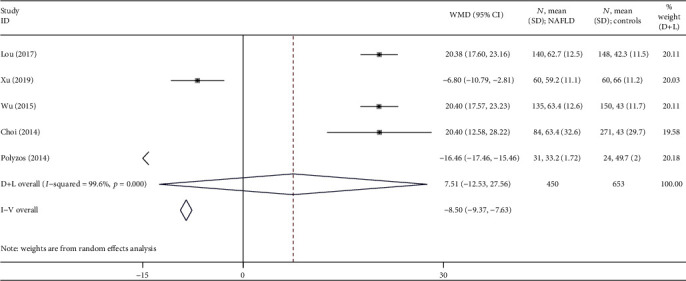
Forest plot analyzing the circulating irisin levels between the NAFLD group and healthy group.

Meta-analyses of the pooled data of 5 case-control studies [15–19] showed that the circulating irisin levels showed no significant differences between the NAFLD group and healthy group (Figure 2). A high heterogeneity (*I*^2^ = 100%, *P* < 0.001) was observed, and so a random effects model was used, showing an overall WMD of 7.51 (-12.53, 27.56) ng/ml with *P* > 0.05.

### 3.4. Subgroup Analysis

**Figure 3:**
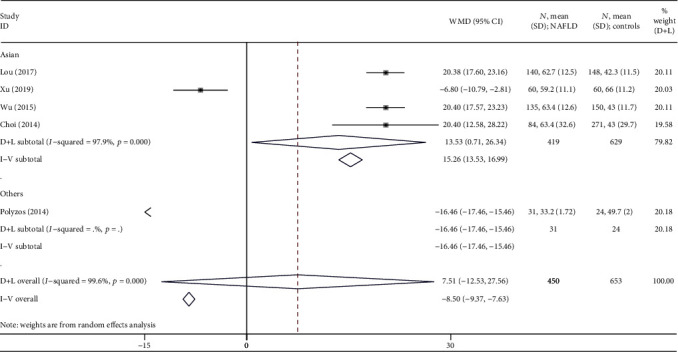
Forest plot analyzing the circulating irisin levels between the NAFLD group and healthy group by races.

Firstly, subgroup analysis was conducted based on the races of people included in this study (Figure 3). The results showed that there were 4 studies conducted in Asians [15–18], and the average irisin levels were higher in the NAFLD group than in the healthy group. A high heterogeneity (${\underset{¯}{I}}^{2}=98\%$, *P* < 0.001) was observed, and so a random effects model was used for calculating the WMD. The overall WMD was 13.53 (0.71, 26.34) ng/ml with *P* < 0.05.

**Figure 4:**
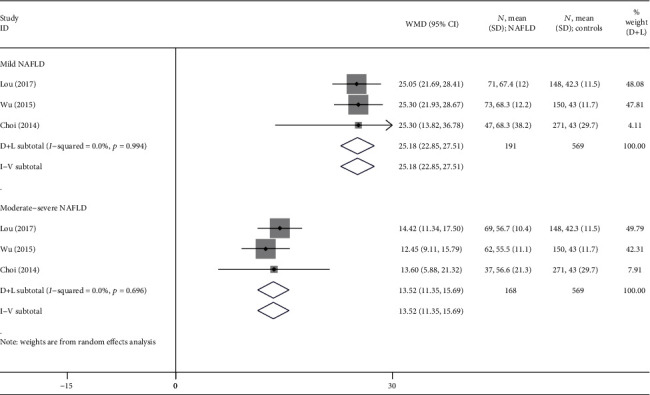
Forest plot analyzing the circulating irisin levels between the mild or moderate-severe NAFLD and healthy groups.

Secondly, subgroup analysis was conducted based on the severity of NAFLD (Figure 4). Meta-analyses were performed for irisin levels between the mild or moderate-severe NAFLD group and healthy group obtained from 3 studies [15–17]. In the mild NAFLD subgroup, the heterogeneity remained low (*I*^2^ = 0%, *P* = 0.99), and so a fixed effects model was used with an overall WMD of 25.18 (22.85, 27.51) ng/ml (*P* < 0.05). In the moderate-severe NAFLD group, the heterogeneity was also low (*I*^2^ = 0%, *P* = 0.70), and so a fixed effects model was used with an overall WMD of 13.52 (11.35, 15.69) ng/ml (*P* < 0.05). The results could be summed up in the mild or moderate-severe NAFLD group, and the average irisin levels were higher in the NAFLD group when compared to the healthy group.

**Figure 5:**
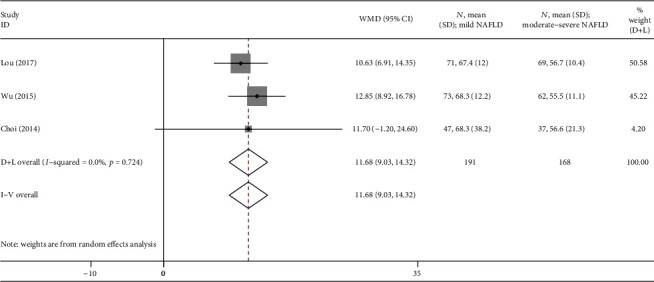
Forest plot analyzing the circulating irisin levels between the mild NAFLD group and the moderate-severe NAFLD group.

Finally, subgroup analysis of 3 studies [15–17] that compared the mild NAFLD group to the moderate-severe NAFLD group was conducted (Figure 5). The results revealed that a high heterogeneity was observed in this subgroup (*I*^2^ = 70%, *P* = 0.03), and so a random effects model was used for calculating the WMD. The overall WMD was 11.68 (9.03, 14.32) ng/ml with *P* < 0.05. The irisin levels were higher in the mild NAFLD group than in the moderate-severe NAFLD group.

### 3.5. Sensitivity Analyses

A significant deviation was detected in the study conducted by Polyzos et al. [19] when analyzing the association of irisin levels between NAFLD patients and healthy controls. After excluding this study, the *P* value showed nonsignificance to significance with increased WMD from 7.51 (-12.53, 27.56) to 13.53 (0.71, 26.34) (Figure 3).

### 3.6. Publication Bias

Egger's tests showed no significant differences in any comparison (*P* > 0.05), indicating a low probability of publication bias.

## 4. Discussion

The purpose of our meta-analysis was to investigate the association of circulating irisin levels in patients with NAFLD. Irisin is a newly discovered muscle/fat factor that is associated with the disturbances in lipid and glucose metabolism or insulin resistance. It is closely related to the occurrence and development of NAFLD.

### 4.1. Theoretical Implications

Fibronectin type III domain-containing protein 5 is the precursor of irisin and is secreted by skeletal muscles into the circulation and plays a role in lipid metabolism. Irisin can increase the expression of uncoupling protein 1 messenger RNA in the adipose tissue. The main role of irisin is to induce brown adipocytes in white adipose tissue depots, which is a process known as white fat “browning,” and this consumes energy to generate body heat [20, 21]. The activated brown adipose tissue consumes excessive fat to generate heat [22]. As a key muscle factor, irisin can connect to the muscle tissue and adipose tissue for crosstalk [23]. The adipose tissue functions to synthesize and secrete irisin, and some scholars have believed that irisin is not only a myokine but also an adipocytokine [24–26].

There are fewer studies with regard to the important mechanisms of irisin in the occurrence of NAFLD now. However, experimental evidence has shown the liver as a target organ of irisin [27]. Hou et al. [28] have conducted a study on obese mice, which showed that exogenous irisin treatment could reduce the body mass and visceral fat levels and improve glucose and lipid metabolic disorder. Zhang et al. [21] have revealed that recombinant irisin in mice administered through intraperitoneal injection has reduced the body weight and improved insulin resistance induced by high-fat diet. Huh et al. [29] have shown that irisin increased adipocyte energy expenditure and the expression of metabolite intermediates and metabolic enzymes, playing a role in inhibiting lipid accumulation. These experimental results showed that irisin has pleiotropic effects on improving the adipocyte metabolism in humans. However, the results of similar experiments were different. Wang et al. [30] have detected the effect of irisin on lipid decomposition of adipocytes after treatment of adipocytes with irisin and showed that irisin does not directly affect the lipidolysis and fatty acid metabolism by adipocytes. There are differences among varied studies conducted, and these might be due to differences in species included, study subjects, and experimental schemes.

### 4.2. Clinical Implications

In recent years, many studies have investigated circulating irisin concentrations in NAFLD subjects, but their conclusions lacked consistency. For example, Shanaki et al. [31] have discovered that the levels of irisin were significantly lower in NAFLD patients when compared to the controls (*P* < 0.001). In contrast, Jiang et al. [32] have studied levels of irisin in liver tissues of NAFLD patients, and their results showed that the irisin levels in NAFLD patients were higher than those in healthy people (66.8 ± 0.45 vs. 42.69 ± 1.00 ng/ml, respectively; *P* < 0.01). Ma et al. [33] have found that the expression of irisin in the liver tissue of patients with NAFLD (56 cases with nonalcoholic simple fatty liver and 75 cases with nonalcoholic steatohepatitis) was higher when compared to that in the healthy control group (1.11 ± 0.63 and 1.17 ± 0.61 vs. 0.58 ± 0.39, respectively; *P* < 0.05). Meanwhile, several studies have been conducted on the relationship between circulating irisin level and disease severity of NAFLD and demonstrated that the irisin level in mild steatosis patients was higher than that in moderate and severe steatosis patients.

The serum irisin levels were increased temporarily during the early stage and then stabilized relatively with the aggravation of the NAFLD process. This might be related to the defense mechanism of the liver in the early period of the disease. There might be resistance to irisin during lipid metabolism. When the severity of NAFLD disease was increased, then the irisin levels could not be increased. From this perspective, irisin might be regarded as a compensatory factor that regulates lipid accumulation in liver cells, and the change of circulating irisin level might act as an independent risk factor for NAFLD.

### 4.3. Limitations

Both English and Chinese language studies were obtained and included to avoid local literature bias. But there are several limitations that could not be neglected.

Firstly, the number of literatures and sample size of the subjects included were not big. Only 5 high-quality studies were included in this meta-analysis. There were many studies on irisin levels in NAFLD patients with diabetes and other diseases combined, but these studies could not be included in this meta-analysis due to not meeting the eligibility criteria. The sample size of the included studies was 120 to 355 from Asia and 39 were from Greece. Subgroup analysis based on race (Figure 3), divided into the Asian subgroup and others (Polyzos et al. from Greece), could also present subgroup analysis based on sample size. Egger's test was performed to test publication bias, and the results indicated a low probability (*P* > 0.05).

Secondly, irisin has been identified as an exercise-induced hormone that is secreted by the skeletal muscles and a muscle factor. Exercise is also a key factor to prevent and manage NAFLD, but there was not enough data to study the relationship between irisin and exercise in patients with NAFLD.

Moreover, recent studies have found that there are many factors that influence different results of irisin, including race [34], gender [35, 36], and age. With limited studies, the results of subgroup analysis by race seemed to show that irisin in patients with NAFLD might be related to race. Sensitivity analyses also demonstrated that racial differences might be one of the factors that contribute to the heterogeneity in this meta-analysis, indicating the associations in Asian populations. Among the included studies, there was only one study [16] that compared irisin levels by gender, and so it is not suitable to be included for subgroup analysis. This study concluded that the serum irisin levels tended to be higher in men than in women and showed significant differences. This suggested that the clinical monitoring of irisin levels in NAFLD patients should also focus on gender differences in further studies.

Finally, subgroup analysis was performed by classifying disease severity with only 3 small sample size studies [15–17] from Asia and evaluated the grades of NAFLD based on abdominal ultrasonography results. These were categorized as a mild fatty liver subgroup and a moderate-to-severe fatty liver subgroup. This suggested that the irisin levels in the mild NAFLD group might be higher than those in the moderate-severe group in Asians. Studies with large sample size from other countries are further warranted to obtain a more robust conclusion.

## 5. Conclusions

Our meta-analyses of 5 case-control studies comprising 1087 participants suggested that the average irisin levels might be higher in the NAFLD group than in the healthy group in Asians. Circulating irisin levels in NAFLD patients might be different based on its severity. The irisin levels in the mild NAFLD group might be higher than those in the moderate-severe group in Asians. It is important to monitor the changing trend of irisin level in predicting the course of NAFLD disease as well as its changes. It is necessary to investigate the causal effect of irisin signaling in the development of NAFLD in the future studies by controlling the refinement of inclusion criteria strictly, including larger sample size, and designing not only more rigorous but also higher-quality randomized controlled trials, thus providing new methods for the treatment and prediction of irisin levels in association with NAFLD.

## Figures and Tables

**Table 1:** Characteristics of studies included in the meta-analysis.

No.	First authors	Year	Country	Numbers		Sex (M/F)		Age		NOS∗	Study design	Type of NAFLD	Grade of NAFLD	Control type
				Cases	Controls	Cases	Controls	Cases	Controls					
1	Lou [17]	2017	China	140	148	97/43	101/47	48.69 ± 8.63	47.36 ± 8.43	5	Case-control	—	Mild Moderate-severe	Healthy control
2	Xu [18]	2019	China	60	60	46/14	42/18	46 ± 8	45 ± 11	6	Case-control	—	—	Healthy control
3	Wu [15]	2015	China	135	150	90/45	103/47	47.87 ± 9.12	48.25 ± 8.16	5	Case-control	—	Mild Moderate-severe	Healthy control
4	Choi [16]	2014	South Korea	84	271	38/46	56/215	48.0 ± 9.8	44.4 ± 9.9	5	Case-control	—	Mild Moderate-severe	Healthy control
5	Polyzos [19]	2014	Greece	15	24	5/10	4/20	53.9 ± 2.6	54.2 ± 1.6	6	Case-control	NAFL+NASH	—	Lean control

^∗^NOS means the Newcastle-Ottawa Scale (NOS).

**Table 2:** Scores of the Newcastle-Ottawa Scale.

No.	First authors	Selection				Comparability	Exposure			Total
		①	②	③	④	⑤	⑥	⑦	⑧	
1	Lou [17]	1	1	0	1	0	1	1	0	5
2	Xu [18]	1	1	0	1	0	2	1	0	6
3	Wu [15]	1	1	0	1	0	1	1	0	5
4	Choi [16]	1	1	0	1	0	1	1	0	5
5	Polyzos [19]	1	1	1	1	0	1	1	0	6

①: is the case definition adequate? ②: representativeness of the cases. ③: selection of controls. ④: definition of controls. ⑤: comparability of cases and controls on the basis of the design or analysis. ⑥: ascertainment of exposure. ⑦: the same method of ascertainment for cases and controls. ⑧: nonresponse rate.

## Data Availability

The retrospective data used to support the findings of this study are included within the article.

## Additional Points

*PRISMA 2009 Checklist Statement*. The authors have read the PRISMA 2009 Checklist, and the manuscript was prepared and revised according to the PRISMA 2009 Checklist.

## Supplementary Materials

Supplementary 1 Supplemental File 1: the published protocol in PROSPERO with the registration number CRD 42019130962.

Supplementary 2 Supplementary File 2: the full search strategy.
