# Supplementary material for: Circulating Irisin Levels in Patients with Nonalcoholic Fatty Liver Disease: A Systematic Review and Meta-Analysis
Source: Gastroenterol Res Pract. 2020 Nov 7;2020:8818191. doi: 10.1155/2020/8818191 (PMC7670588; doi:10.1155/2020/8818191)
Supplement: Supplementary 1 — Supplemental File 1: the published protocol in PROSPERO with the registration number CRD 42019130962. [file 8818191.f1.pdf]

# Circulating irisin level in patients with nonalcoholic fatty liver disease: a systematic review and meta-analysis

*Jie Hu, Shan Liu*

## Citation

Jie Hu, Shan Liu. Circulating irisin level in patients with nonalcoholic fatty liver disease: a systematic review and meta-analysis. PROSPERO 2019 CRD42019130962 Available from:
https://www.crd.york.ac.uk/prospero/display_record.php?ID=CRD42019130962

## Review question

Studies measuring circulating irisin levels in patients with Nonalcoholic Fatty Liver Disease have achieved controversial results. Our systematic review and meta-analysis aims to assess the circulating irisin levels in patients with Nonalcoholic Fatty Liver Disease. Moreover, the secondary aim is to analyze the relationship between irisin and blood lipid in patients with Nonalcoholic Fatty Liver Disease.

## Searches

We will search through PubMed, EMBASE, Cochrane Library, WANFANG, CNKI and CBM databases, using the terms “fatty liver” (OR “NAFLD” OR “NASH” OR “nonalcoholic steatohepatitis” OR “nonalcoholic fatty liver disease”) AND "irisin" or "FNDC5". Moreover, the reference lists in previously published reviews and original research articles will be scrutinized to identify publications not covered by the original database searches. No language limitations will be used. In addition, we will contact study authors by e-mail to identify additional studies and ask for missing data.

## Types of study to be included

Studies will be included if they are case-control studies or cohort studies published that report data on circling irisin level in individuals with/without NAFLD.

## Condition or domain being studied

Nonalcoholic fatty liver disease (NAFLD), is a global public health problem of increasing significance, ranges from simple nonalcoholic fatty liver (NAFL) to nonalcoholic steatohepatitis(NASH), characterized by steatosis, inflammation and fibrosis. NASH, a severe form of NAFLD, may lead to subacute liver failure, liver cirrhosis and/or hepatocellular carcinoma.

## Participants/population

We will include only studies conducted in adults (aged 18 years or older) in which NAFLD was diagnosed either by imaging or by histology. Exclusion criteria included secondary hepatic fat accumulation, such as significant alcohol consumption, use of steatogenic medication, or hereditary disorders, other known causes of liver diseases, e.g. virus and drugs.

## Intervention(s), exposure(s)

Individuals with NAFLD, Circulating irisin level , Blood lipid index

## Comparator(s)/control

Control cases were healthy people.

## Context

## Main outcome(s)

The most important outcome is circulating irisin levels measured by enzyme-linked immunosorbent assay

(ELISA) in either plasma or serum .

## Additional outcome(s)

The relationship between irisin and blood lipid indexes in patients with Nonalcoholic Fatty Liver Disease.

## Data extraction (selection and coding)

Data extraction will be performed independently by two investigators according to the inclusion criteria. The third participant will be consulted for discussion to reach agreement concerning discrepancies. The following items will be extracted from each study: first author's last name, publication date, country of origin, the Newcastle-Ottawa Scale (NOS), numbers of cases and controls, irisin levels measured method, levels of irisin in NAFLD group/ control group, levels of blood lipid indxes in NAFLD group/ control group.

## Risk of bias (quality) assessment

Quality of the included articles will be evaluated using Newcastle-Ottawa Scale (NOS) scale. The NOS contains eight items categorized into three dimensions including selection, comparability, and exposure. For each item a series of response options is provided. A star system is used to allow a semi-quantitative assessment of study quality, such that the highest quality studies are awarded a maximum of one star for each item with the exception of the item related to comparability that allows the assignment of two stars. The NOS ranges between zero and nine stars.

## Strategy for data synthesis

Review manager 5.3 and Stata 15 software will be used for statistical analysis to perform meta-analysis. Heterogeneity will be checked by the $?^2$ test (Cochran 1954) and the $I^2$ statistic (Higgins 2003). The criteria for identification of heterogeneity will be a P value less than 0.10 for the $?^2$ test and an $I^2$ statistic greater than 50%. When there is no statistical evidence for heterogeneity in effect sizes, we will use the fixed-effect model (Mantel 1959). When significant heterogeneity is identified, we will use the random-effects model (DerSimonian 1986) and will explore sources of significant heterogeneity by subgroup analysis or meta-regression.

## Analysis of subgroups or subsets

Subgroup analyses will be performed by ethnicity.

## Contact details for further information

Jie Hu
muhudie1106@163.com

## Organisational affiliation of the review

The First Affiliated Hospital of Zhejiang Chinese Medical University

## Review team members and their organisational affiliations

Dr Jie Hu. The First Affiliated Hospital of Zhejiang Chinese Medical University
Dr Shan Liu. The First Affiliated Hospital of Zhejiang Chinese Medical University

## Type and method of review

Meta-analysis, Systematic review

## Anticipated or actual start date

08 April 2019

## Anticipated completion date

01 July 2019

## Funding sources/sponsors

The Natural Science Foundation of Zhejiang Province, China, No. LQ19H290001.

## Conflicts of interest

None known

## Language

English

## Country
China

## Stage of review
Review Ongoing

## Subject index terms status
Subject indexing assigned by CRD

## Subject index terms
Fibronectins; Humans; Non-alcoholic Fatty Liver Disease; Risk Factors

## Date of registration in PROSPERO
08 May 2019

## Date of publication of this version
08 May 2019

## Details of any existing review of the same topic by the same authors

## Stage of review at time of this submission
The review has not started

| Stage | Started | Completed |
|---|---|---|
| Preliminary searches | No | No |
| Piloting of the study selection process | No | No |
| Formal screening of search results against eligibility criteria | No | No |
| Data extraction | No | No |
| Risk of bias (quality) assessment | No | No |
| Data analysis | No | No |

## Versions
08 May 2019

PROSPERO
This information has been provided by the named contact for this review. CRD has accepted this information in good faith and registered the review in PROSPERO. The registrant confirms that the information supplied for this submission is accurate and complete. CRD bears no responsibility or liability for the content of this registration record, any associated files or external websites.
