# Supplementary material for: Circulating Irisin Levels in Patients with Nonalcoholic Fatty Liver Disease: A Systematic Review and Meta-Analysis
Source: Gastroenterol Res Pract. 2020 Nov 7;2020:8818191. doi: 10.1155/2020/8818191 (PMC7670588; doi:10.1155/2020/8818191)
Supplement: Supplementary 2 — Supplementary File 2: the full search strategy. [file 8818191.f2.docx]

Supplemental File 2. Search strategy

| **Databases** | **Search terms** | **Numbers of records** |
| --- | --- | --- |
| **Pubmed** | Search((((("Non-alcoholic Fatty Liver Disease"[Mesh]) OR ((NASH OR Nonalcoholic fatty liver disease OR Non alcoholic fatty liver disease OR Non alcoholic Fatty Liver Disease OR NAFLD OR Nonalcoholic Fatty Liver Disease OR Nonalcoholic Fatty Liver OR Nonalcoholic Steatohepatitis OR Nonalcoholic Steatohepatitides)))) OR ((fatty liver OR Liver, Nonalcoholic Fatty OR Steatohepatitides, Nonalcoholic OR Steatohepatitis, Nonalcoholic)))) AND ((("irisin protein, zebrafish" [Supplementary Concept]) OR "FNDC5 protein, human" [Supplementary Concept]) OR ((irisin OR FNDC5 OR fibronectin type III domain containing protein 5 OR Fndc5 protein OR FRCP2 protein))) | 43 |
| **Cochrane Library** | #1 MeSH descriptor:[Non-alcoholic Fatty Liver Disease] explode all trees  #2 fatty liver or Liver, Nonalcoholic Fatty or Steatohepatitides, Nonalcoholic or Steatohepatitis, Nonalcoholic or NASH or Nonalcoholic fatty liver disease or Non alcoholic fatty liver disease or Non alcoholic Fatty Liver Disease or NAFLD or Nonalcoholic Fatty Liver Disease or Nonalcoholic Fatty Liver or Nonalcoholic Steatohepatitis or Nonalcoholic Steatohepatitides  #3 irisin or FNDC5 or fibronectin type III domain containing protein 5 or Fndc5 protein or FRCP2 protein  #4 #1 OR #2  #5 #4 AND #3 | 9 |
| **EMBASE** | #1 'nonalcoholic fatty liver'/exp OR 'nonalcoholic fatty liver'  #2 'fatty liver' OR 'liver, nonalcoholic fatty' OR 'steatohepatitides, nonalcoholic' OR 'steatohepatitis, nonalcoholic' OR 'nash' OR 'non alcoholic fatty liver disease' OR 'nafld' OR 'nonalcoholic fatty liver disease' OR 'nonalcoholic fatty liver' OR 'nonalcoholic steatohepatitis' OR 'nonalcoholic steatohepatitides'  #3 'fndc5 protein'/exp OR 'fndc5 protein'  #4 'irisin'/exp OR 'irisin' OR 'fibronectin type iii domain containing protein 5'/exp OR 'fibronectin type iii domain containing protein 5'  #5 'irisin' OR 'fndc5' OR 'fibronectin type iii domain containing protein 5' OR 'fndc5 protein' OR 'frcp2 protein'  #6 #1 OR #2  #7 #3 OR #4 OR #5  #8 #6 AND #7 | 71 |
| **CNKI** | FT=('nonalcoholic fatty liver'+'nonalcoholic fatty liver disease'+'nonalcoholic steatohepatitis'+'nonalcoholic hepatitis'+'Steatohepatitis'+'nonalcoholic Simple fatty liver') and FT=('irisin'+'FNDC5'+' fndc5 protein'+' fibronectin type iii domain containing protein 5') | 383 |
| **Wangfang** | ( nonalcoholic fatty liver+nonalcoholic fatty liver disease+nonalcoholic steatohepatitis+nonalcoholic hepatitis+Steatohepatitis+nonalcoholic Simple fatty liver) * (irisin+FNDC5+fndc5 protein+fibronectin type iii domain containing protein 5) | 38 |
